# Efficacy of Transosseous Tunnel Placement for Triple Endobutton Plate in Acromioclavicular Joint Reconstruction: A Three‐Dimensional Printing Guide Design Technology

**DOI:** 10.1111/os.13091

**Published:** 2021-12-13

**Authors:** Lei Zhang, Youliang Wen, Meng‐yao Zhang, Xin Zhou, Shi‐jie Fu, Guo‐you Wang

**Affiliations:** ^1^ Department of Orthopedics Affiliated Traditional Chinese Medicine Hospital of Southwest Medical University Luzhou China; ^2^ Center for Orthopedic Diseases Research Affiliated Traditional Chinese Medicine Hospital of Southwest Medical University Luzhou China; ^3^ Expert Workstation in Luzhou Luzhou China; ^4^ Clinical Base of Affiliated Traditional Chinese Medicine Hospital of Southwest Medical University Guangdong Province Medical 3D Printing Application Transformation Engineering Technology Research Center Luzhou China; ^5^ School of Rehabilitation Medicine GanNan Medical University Ganzhou China; ^6^ School of Clinical Medicine Southwest Medical University Luzhou China

**Keywords:** Acromioclavicular joint dislocation, Guide design, Three‐dimensional printing, Transosseous tunnel placement, Triple Endobutton

## Abstract

**Objective:**

Explore an accurate transosseous tunnel drilling method based on three‐dimensional (3D) printing technology for acromioclavicular joint reconstruction (ACD), design a guide design, and evaluate its accuracy.

**Methods:**

Using Mimics software to reconstruct 100 cases of acromioclavicular joint computed tomography (CT) data. In design 2, the non‐collinear tunnel is superimposed on the 3D model, and a virtual drilling is performed between the clavicle and the coracoid using a triple inner gusset. Then, in the Geomagic Studio software model, an elliptical plane is calculated and extracted as a guide design for precise drilling. Then put the design and the 3D shoulder model together for 3D printing. Ten lengths were measured, and the effects of the virtual model, the actual model, and the guide rail design were compared.

**Results:**

We successfully compared 10 parameters of 3D virtual model and actual model. There was no significant difference between actual and virtual bone tunnels in 10 measurements (*P* > 0.05).

**Conclusions:**

The accuracy of ACD combined with 3D printing guidance design technology in the transosseous tunnel of adult shoulder is reliable.

## Graphical abstract

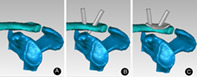

## Introduction

Acromioclavicular joint consists of the lateral clavicle and medial acromion, which is an amphiarthrosis^1^. Many biomechanical studies have confirmed that the surrounding ligaments and muscles are critical to its stability and movement, especially its main stable structure, the acromioclavicular ligament, including the trapezoidal ligament and conical ligament^2^, ^3^. Acromioclavicular dislocation (ACD) is a common shoulder injury, and the incidence rate is about 9%–12%^4^. The pathogenesis of ACD is usually direct impact on shoulder adduction, leading to downward dislocation of the glenoid and clavicle impaction on the first rib^5^. Acromioclavicular ligaments are the initial damages, then it ranges from a simple sprain of acromioclavicular ligament to a complete dislocation of the joint if not treated at the right time. Nowadays, due to the high incidence of ACD and the increased risk of re‐dislocation, it has become a research hotspot. Based on the anatomical feature of acromioclavicular joint, four types were divided, Rockwood I ~ IV^4^, ^6^. As we all known, ACD does not only cause shoulder pain and abnormal movement, but also affects the movement of the whole upper limbs, affecting the patient's normal life. Therefore, reconstruction of the acromioclavicular joint is a top priority.

Recently, acromioclavicular joint reconstruction techniques have recently focused on anatomical restoration of the coracoclavicular ligaments. Many articles reported the triple Endobutton plate to treat the complete ACD^4^, ^7^, ^8^, ^9^, ^10^. This individually reestablishes the conoid and trapezoid ligaments using three button plates and two strand fiber sutures. Two transosseous tunnels need to be drilled at the distal end of the clavicle to reconstruct the complete ACD. Accurately finding the drilling point is essential to reduce iatrogenic complications such as beak fractures. Maziak^11^ reported that triple Endobutton plate led to a satisfactory clinical result and provided excellent biomechanical stability. Although the triple Endobutton technology has solved a lot of problems, there are still some shortcomings, for example the length of the fiber suture cannot be adjusted at will. Although the triple Endobutton technology has solved many problems, there are still some shortcomings, such as the length of the fiber suture cannot be adjusted at will. With the increasing development of three‐dimensional (3D) printing technology, some orthopaedic surgeons gradually apply it to clinical work to give patients a specific treatment without causing too much trauma, because it could provide anatomical details of various bones and tissues^12^, ^13^. At present, the use of 3D printing technology to process ACD has made some significant progress, creating individualized guided design and trying to determine the ideal location of the transosseous tunnel^14^, ^15^, ^16^. As far as we know, guided design can quickly and accurately find the correct position, and improve the feasibility, safety, accuracy, and effectiveness of ACD reconstruction surgery.

Hence in this study, we compared the difference between 3D virtual model and actual model, combined with the lead design, and used the triple Endobutton plate to reconstruct the disordered acromioclavicular joint, aiming to accurately find the subject‐specific transosseous tunnel. This study verified the feasibility, safety, and accuracy of the lead design, and provided theoretical support and experimental basis for clinical reconstruction of ACD.

The purpose of this study was: (i) to explore the accuracy of 3D printing triple Endobutton technique, reducing complications; (ii) to compare the differences of 3D virtual model and actual model, verifying the credibility of the application of 3D printing triple Endobutton technique in surgery.

## Materials and Methods

### Patients

We collected 160 normal shoulder computed tomography (CT) scan data from April 2019 to December 2019. Exclusion criteria include shoulder fracture, dislocation, and implantation. We selected 100 neat and complete shoulder joints (50 on the left and 50 on the right) from 160 cases for digital imaging and medical communication (DICOM) data.

### The Design of 3D Printing Guide Design

**Fig. 1:**
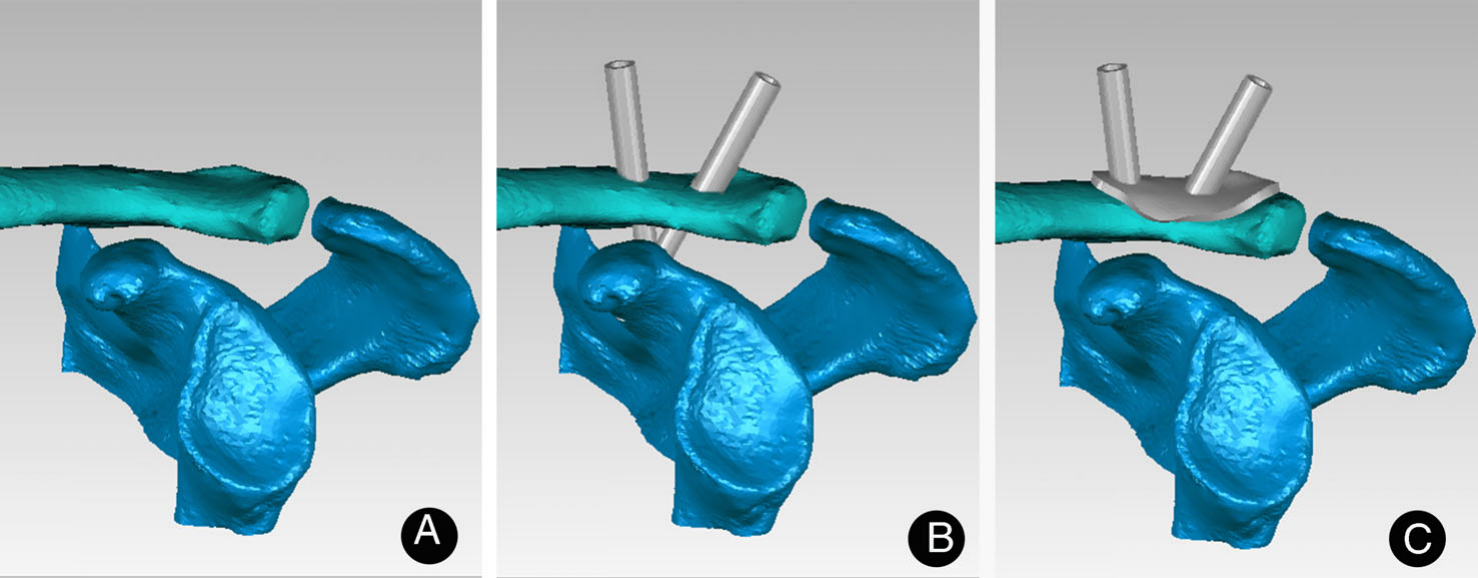
Establishment of guide design module (A) acromioclavicular joint reconstruction model; (B) two transosseous tunnels for triple Endobutton technology in 3D reconstruction acromioclavicular joint model; (C) acromioclavicular joint reconstruction model with guide design for triple Endobutton technology.

All DICOM data were imported into the image processing software (Mimics 19.0) to construct virtual 3D shoulder models. Then we retained the clavicle and scapula to monitor and virtually drill the transosseous tunnel. Based on some anatomical parameters, we identified the optimum drilling points, respectively, and then used two non‐collinear hollow cylinders (Interior Radius 2 mm and Laterior Radius 4 mm, respectively) superimposed on the 3D models to represent a virtual drilling tunnel between clavicle and coracoid. Meanwhile, we imported the 3D models into the software (Geomagic Studio 2013.0) to calculate and extract an oval plane (the thickness, 2.5 mm, and the direction, upward) surrounding the tunnel along the clavicle as guide design for accurately drilling in the surgery to reconstruct acromioclavicular joint. We then put the design and 3D shoulder model together in Mimics 19.0 (Fig. 1). The data of the combination were transformed into print file of Replicator Z18 printer by MakerBot Print software (printing parameters: print mode, balance; layer height, 0.2 mm; wall thickness, two times the thickness of sprinkler head; sprinkler moving speed, 150 mm/s; sprinkler temperature, 215°C; sprinkler wire diameter, 1.77 mm; the platform withdrawal height, 0.5 mm; the top thickness, 0.804 mm; the bottom thickness, 0.8 mm; the minimum supporting angle, 68°; the supporting density, 16%; and the printing material: biodegradable plastic polylactic acid).

### The Parameters of 3D Printing Guide Design

**Fig. 2:**
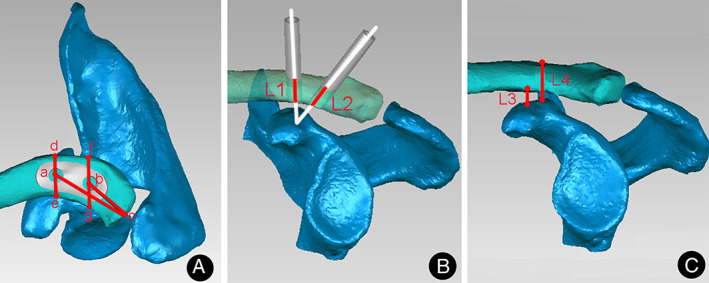
The diagram of 3D shoulder model (A) The top view of acromioclavicular joint reconstruction model; (B) The inside view of acromioclavicular joint with two transosseous tunnels; (C) The inside view of acromioclavicular joint reconstruction model. ac: the distance from the center of anterior clavicle tunnel to the distal acromioclavicular joint; bc: the distance from the center of interior clavicle tunnel to the acromioclavicular joint; ad: the closest distance between the center of anterior clavicle tunnel and the posterior border of clavicle; ae: the closest distance between the center of anterior clavicle tunnel and the anterior border of clavicle; bf: the distance closest between the center of interior clavicle tunnel and the posterior border of clavicle; bg: the closest distance between the center of interior clavicle tunnel and the anterior border of clavicle; L1: the length of lateral clavicle tunnel; L2: the length of interior clavicle tunnel; L3: the closest distance from superior coracoid to inferior clavicle; L4: the closest distance from superior coracoid to superior clavicle.

Reference point (a): the center of lateral clavicle tunnel; (b): the center of interior clavicle tunnel; (c): acromioclavicular joint; (d, f): the posterior of clavicle; (e, g): the anterior of clavicle. At last, 10 lengths were measured to compare the effect of the virtual model and actual model with the guide design (Fig. 2). The virtual tunnel can be measured and recorded with an accuracy of up to 0.1 mm in Mimics 19.0. To avoid observer variation, an investigator with over 2 years of experience in 3D printing work measured and recorded 10 parameters carefully three times, before taking the average.

1. ac: the distance from the center of anterior clavicle tunnel to the distal acromioclavicular joint.
2. bc: the distance from the center of interior clavicle tunnel to the acromioclavicular joint.
3. ad: the closest distance between the center of anterior clavicle tunnel and the posterior border of clavicle.
4. ae: the closest distance between the center of anterior clavicle tunnel and the anterior border of clavicle.
5. bf: the distance closest between the center of interior clavicle tunnel and the posterior border of clavicle.
6. bg: the closest distance between the center of interior clavicle tunnel and the anterior border of clavicle.
7. L1: the length of lateral clavicle tunnel.
8. L2: the length of interior clavicle tunnel.
9. L3: the closest distance from superior coracoid to inferior clavicle.
10. L4: the closest distance from superior coracoid to superior clavicle.

### Statistical Analysis

Measurement data were expressed as mean ± standard deviation (*x*¯*±s*) in mm. A paired two‐tailed t‐test was performed to identify differences between measurements in 3D virtual model and actual model if the data were normally distributed. Statistical analysis was performed using the SPSS22.0 (IBM Corp., Armonk, NY, USA); *P*‐value <0.05 was considered statistically significant.

## Results

We have printed 100 human shoulders successfully. Then 10 parameters of the 3D virtual model and actual model with the guide design were measured and compared in Fig. 2. We found that there was no difference between the actual and virtual bone tunnels in ac, bc, ad, ae, bf, bg, L1, L2, L3, L4 (*P* > 0.05). All data were represented as*x*¯*±s* in Table 1.

**TABLE 1:** The parameters of the 3D virtual model and actual model (*x*¯*±s*)

Parameter	3D virtual model	Actual model	t‐value	*P*‐value
ac (mm)	32.73 ± 0.29 *	32.43 ± 0.22	12.941	0.231
bc (mm)	4.43 ± 0.41 *	4.36 ± 0.37	9.018	0.150
ad (mm)	11.63 ± 0.52 *	11.57 ± 0.58	7.018	0.135
ae (mm)	11.45 ± 0.51 *	11.40 ± 0.66	19.814	0.357
bf (mm)	12.13 ± 0.29 *	12.08 ± 0.36	1.987	0.849
bg (mm)	11.89 ± 0.32 *	11.96 ± 0.34	4.018	0.131
L1 (mm)	8.73 ± 0.52 *	8.66 ± 0.31	8.081	0.241
L2 (mm)	8.97 ± 0.62 *	9.03 ± 0.70	0.973	0.083
L3 (mm)	8.46 ± 0.20 *	8.42 ± 0.18	2.018	0.093
L4 (mm)	16.29 ± 0.67 ^*^	16.35 ± 0.79	32.091	0.983

The 3D virtual model was made with Geomagic Studio. The actual model was the 3D model printed with MakerBot Print.

* *P >* 0.05 *vs* actual model.

## Discussion

The acromioclavicular joint is a joint stabilized by many ligaments and muscles^1^, ^3^. Biomechanics and anatomical section studies have shown that the coracoclavicular ligament has different functions under different directions and forces^3^. People who use their arms for a long time, especially athletes, are more susceptible to ACD. The mechanism of ACD is usually to directly hit the adducted shoulder, causing downward dislocation of the scapula opposite to the impact of the clavicle^5^, ^17^. Firstly, the acromioclavicular ligament is injured, and then from simple acromioclavicular ligament injury to complete dislocation of the joint, if the best time for treatment is delayed. ACD is a common shoulder joint injury, accounting for about 9% ~ 12%. Due to the high incidence of ACD and the increased risk of re‐dislocation, ACD has become a research hotspot. According to the anatomical characteristics of the acromioclavicular joint, we divide the acromioclavicular joint into four types: Rockwood I~IV^4^, ^5^, ^17^. As we all know, ACD not only causes acromioclavicular joint pain and abnormal movement, but also affects the limb movement of the entire upper limb and affects the normal life of the patient.

Nonoperative treatments are recommended for Rockwood I and II separations^18^. Since Podgorski^1^ adopted surgical treatment for ACD in 1987, the treatment Rockwood III and IV have still been controversial. Overall, more than 60 surgical techniques have been described, but there is no gold standard of the surgical management of high‐grade ACD^19^. Operative treatments mainly incorporate acromioclavicular joint reduction and internal fixation, the transposition procedure of dynamic muscle, and reconstruction and fixation of the ligament of acromioclavicular joint^11^, ^20^. Some scholars use various forms of hardware fixation, such as Bosworth screws, but this treatment is no longer popular because the hardware requires a second operation, leading to the reported high failure rate^4^, ^19^, ^20^, ^21^. Other scholars believe that the internal fixation is only a means of short‐term functional recovery. In that case, clinicians began to shift their attention to research using a novel approach. Struhl^22^ first applied Endobutton plate ligament reconstruction beak lock for ACD in 2007, which significantly reduced postoperative complications. Recently, the use of triple Endobutton can repair the coracoclavicular ligament in an anatomical location, with stronger damage characteristics. This technology has good biomechanical stability, good treatment effect, small trauma, and reduces postoperative pain and postoperative reoperation^5^, ^7^, ^8^, ^9^. As is known to all, the difficulty of the triple internal buckle technique is to establish a transosseous tunnel at the clavicle and coracoid process to accurately reconstruct the normal anatomical structure of the coracoclavicular ligament. Determining the ideal, safe, and accurate tunnel location is crucial.

Although the triple Endobutton technology has solved a lot of problems, recent clinical studies have reported the complication rate of the coracoclavicular ligament reconstruction technique is as high as 23% to 80%. There are still some shortcomings, for example the length of the fiber suture cannot be adjusted at will; and the potential risk of clavicle and/or coracoid process fractures, owing to the degree of transosseous tunnel and surgical complexity. At the same time, the triple Endobutton technology uses two cross‐bone tunnels at the distal end of the clavicle, which increases the risk of fracture of the clavicle process^4^, ^19^, ^21^. In order to better maintain the horizontal stability of the acromioclavicular joint and reduce complications, we should be carefully positioned he relative orientation of the transosseous tunnel, which is the angle of the drill tunnel should be aligned with the coracoclavicular ligament.

In fact, he location and method of drilling through the bone tunnel on the coracoid process or the clavicle vary from operator to operators^5^, ^22^. In order to determine the ideal transosseous tunnels between the clavicle and the coracoid process, many studies have researched the anatomical characteristic of coracoclavicular ligament. In that case, our research designed an individualized 3D printed guide design, which is placed on the exposed clavicle helping to find the precise anatomical location of the transosseous tunnel drilling during the operation^12^, ^13^, ^23^. At the same time, compared with conventional operation, this method can improve the accuracy of drilling and reduce the risk of complications. Many studies revealed that 3D printing technology requires less operating time^24^. The results show that there is no difference between the actual model and the virtual model of the ACD using triple Endobutton. It is reliable that an actual model with individualized 3D printed guide design improves the accuracy of transosseous tunnel, preventing the destruction of the cortex of the clavicle and coracoid^12^, ^25^, ^26^. Moreover, our research provides theoretical support and experimental basis for triple Endobutton plate to be popularized in clinical practice.

### Limitations

Our study has some weaknesses. First, the samples and individualized 3D printed guide design are imitated in this study. These prospective CT scans do not consider whether the AC joint itself affects the building of the 3D guide design, which may lead to observational errors. Finally, this study is an *in vitro* research, and its rationality and availability need to be confirmed by further studiess in clinical trials. More studies should perform analysis of using triple Endobutton to treat ACD *in vivo* and are conducive to the long‐term clinical outcome.

### Conclusion

In conclusion, there is no difference between the 3D virtual model reconstructed by ACD and the actual model. The individualized 3D guide design for acromioclavicular joint can improve the accuracy of transosseous tunnels and decrease the risk of the complications. The accuracy of ACD combined with 3D printing guidance design technology in the transosseous tunnel of adult shoulder is reliable.

## Conflict of Interest

The authors would like to thank all patients who agreed to participate in this study and the Affiliated Traditional Chinese Medicine Hospital of Southwest Medical University for providing the PACS CT system.

## Authors' Contributions

Lei Zhang: conception and design, editing, and processing of articles. Youliang Wen: picture data processing and statistical analysis. Mengyao Zhang: conducting experiments and data collection. Xin Zhou: conception, design, and statistical analysis. Shi‐jie Fu: conducting experiments. Guo‐you Wang: conception and design.

## Ethics Approval

All procedures were allowed by the Medical Ethics Review Board of Affiliated Traditional Chinese Medicine Hospital of Southwest Medical University with the following reference number: KY2018032.
